# Serous Retinal Detachment Associated with Dome-Shaped Macula and Staphyloma Edge in Myopic Patients before and after Treatment with Spironolactone

**DOI:** 10.1155/2016/8491320

**Published:** 2016-01-28

**Authors:** Álvaro Fernández-Vega Sanz, Carlos Mario Rangel, Eva Villota Deleu, Beatriz Fernández-Vega Sanz, Ronald Mauricio Sánchez-Ávila

**Affiliations:** ^1^Instituto Oftalmológico Fernández-Vega, 33012 Oviedo, Spain; ^2^Instituto Universitario Fernández-Vega, Universidad de Oviedo, Oviedo, Spain; ^3^Fundación Oftalmológica de Santander (FOSCAL), Floridablanca 681004, Colombia; ^4^Universidad Industrial de Santander, Bucaramanga 680002, Colombia; ^5^Hospital Universitario Central de Asturias, Oviedo, Spain

## Abstract

*Objective*. Serous retinal detachment (SRD) is a common anatomical complication associated with dome-shaped macula (DSM) and staphyloma margin in myopic patients. Here we described the anatomical and functional outcomes obtained with the use of oral spironolactone, a mineralocorticoid antagonist, in the management of myopic patients with SRD associated with DSM and staphyloma margin.* Methods*. We evaluated both eyes of twelve myopic patients with long-standing SRD associated with DSM or staphyloma margin. The patients were treated daily for six months with oral spironolactone 50 mg. Best-corrected visual acuity (BCVA) and central retinal thickness (CRT), determined by optical coherence tomography, were evaluated on the first day and on monthly follow-up visits.* Results*. Pretreatment BCVA (mean ± standard deviation) was 0.406 ± 0.324 LogMAR, and posttreatment BCVA was 0.421 ± 0.354 LogMAR (*P* = 0.489). Pretreatment CRT was 323.9 ± 78.6 *μ*m, and after six months of treatment it was significantly lower, 291.2 ± 74.5 *μ*m (*P* = 0.010). There were no treatment-related complications.* Conclusions.* We evaluated a novel treatment for SRD associated with DSM and staphyloma margin in myopic patients. After six months of treatment with the mineralocorticoid antagonist spironolactone, the subretinal fluid and CRT were significantly reduced; however, there was no improvement in BCVA.

## 1. Introduction

Dome-shaped maculas were described by Gaucher et al. as convex elevations of the macula within a myopic staphyloma [1]. Multiple theories have been proposed to explain the pathophysiology, but to date no clear etiology has been established. In addition to dome-shaped maculas, type V staphylomas were characterized by Curtin [2] as changes in the curvature radius of the eyeball on the edge of a staphyloma. If this edge affects the macular area, it can lead to anatomic and visual disturbances. These two entities share the same characteristic convex elevation of the eye wall. Using optical coherence tomography (OCT), Coco et al. described this deformation as “macular bending” [3]. These morphological alterations can cause a variety of complications during their natural course of development. Staphyloma edges have been described for serous retinal detachment (SRD), choroidal neovascularization (CNV), polypoidal choroidal vasculopathy, and atrophy of the retinal-pigmented epithelium (RPE) [4]. Among these, SRD is the most frequent complication. In the case of dome-shaped macula, we have found the following complications: SRD, CNV, extrafoveal schisis, foveoschisis, lamellar macular hole [5], and full-thickness macular hole [3]. Again, SRD is the most common complication.

Currently there is no adequate treatment for SRD associated with a dome-shaped macula or staphyloma edge. There have been several approaches, ranging from observation [6] in which spontaneous resolution occurred in some cases [7] to other treatments such as argon laser photocoagulation [1], photodynamic therapy [8], and intravitreal antiangiogenic therapy with inhibitors of vascular endothelial growth factor [9]. The results have been highly variable. Recently, Dirani et al. reported clinical and anatomical improvement with the use of oral spironolactone in two patients with SRD associated with dome-shaped macula [10]. Thus the aim of our study was to describe the results of spironolactone treatment of myopic patients with SRD associated with a dome-shaped macula or a staphyloma edge.

## 2. Materials and Methods

We performed a prospective, nonrandomized, sequential recruitment of potential subjects at a single center. Both eyes of 12 myopic patients seen in consultation from July 2014 to February 2015 were evaluated. The study was approved by the ethics committee of the institution. This study adheres to the principles of the Declaration of Helsinki, and all patients signed the informed consent. Only myopic patients with SRD associated with a dome-shaped macula or a staphyloma margin were included. Patients with pathology of the cornea, cataract, glaucoma, retinal detachment, optic neuropathy, or atrophic, neovascular, or tractional myopic maculopathy were excluded.

All patients underwent a thorough eye examination that included measurement of visual acuity with Snellen charts recorded as the logarithmic minimum angle of resolution (LogMAR), slit-lamp biomicroscopy, intraocular pressure with a Goldmann tonometer, and fundoscopy under pupil dilatation. On the first visit, imaging of all patients included photography of the ocular fundus (TRC50LX, Topcon Corp., Tokyo, Japan), OCT with 5 scan lines of 6 mm in length, spaced 0.25 mm, rotated in the horizontal, oblique, and vertical meridians (HD-5 Line Raster-Adjustable Cirrus OCT, Carl Zeiss Meditec, Inc., Dublin, CA, USA), and ultrasound (Ocuscan, Alcon, Fort Worth, TX, USA; Cinescan, Quantel Medical SA, Clermont-Ferrand, France) to measure axial length.

Patients were given oral spironolactone 50 mg daily for 6 months, and fundus photography and OCT were repeated monthly. Blood potassium levels were monitored and the patients were questioned about potential side effects.

Descriptive statistics were performed to determine the distributions of absolute and relative frequencies for qualitative variables and means and standard deviations for quantitative variables (SPSS v20.0 for Windows software, SPSS Inc., Chicago, IL, USA). The distribution normality was determined by the Kolmogorov-Smirnov test for each sample of each variable analyzed. Any potential differences observed between basal and final best-corrected visual acuity (BCVA) and central retinal thickness (CRT) were analyzed using the Wilcoxon nonparametric statistical test. The level of statistical significance was set at *P* < 0.05.

## 3. Results

**Figure 1:**
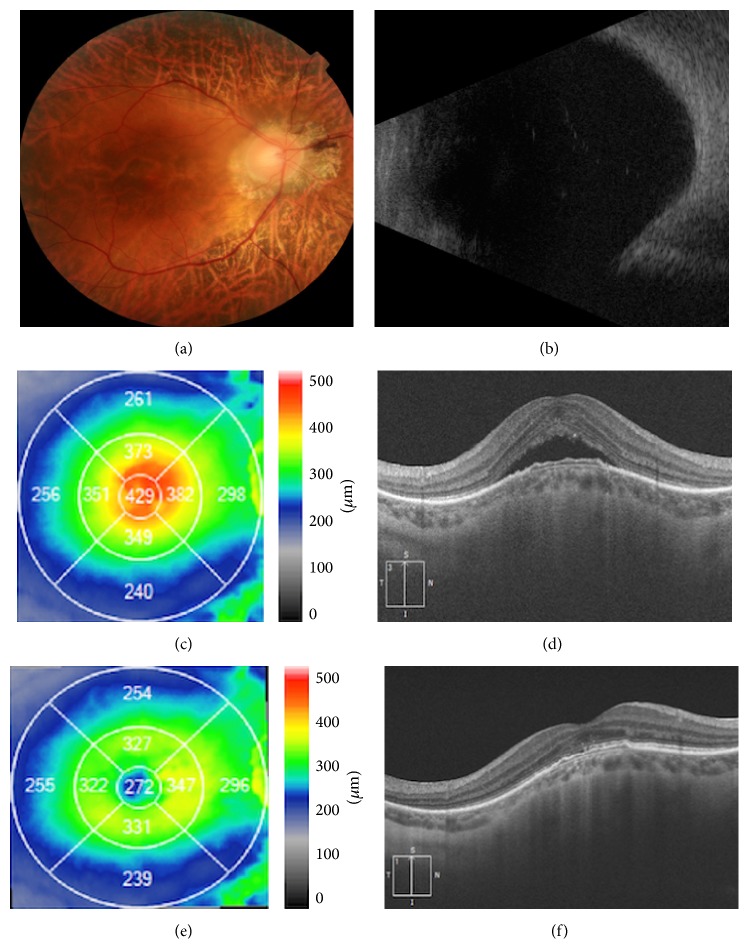
Dome-shaped macula with serous retinal detachment before and after treatment with spironolactone. (a) Color photo showing peripapillary atrophy, marked retinal thinning with visible choroidal vessel, but no hemorrhage in the foveal area. (b) B-scan ultrasound showing an abnormal ocular wall in the posterior fundus. (c) Pretreatment OCT macular map showing increased CRT. (d) Pretreatment OCT image showing serous retinal detachment in the foveal area with no other alterations. (e) Posttreatment OCT macular map showing improved CRT. (f) Posttreatment OCT image showing resolution of the serous retinal detachment in the foveal area with an abnormal ellipsoid line.

**Figure 2:**
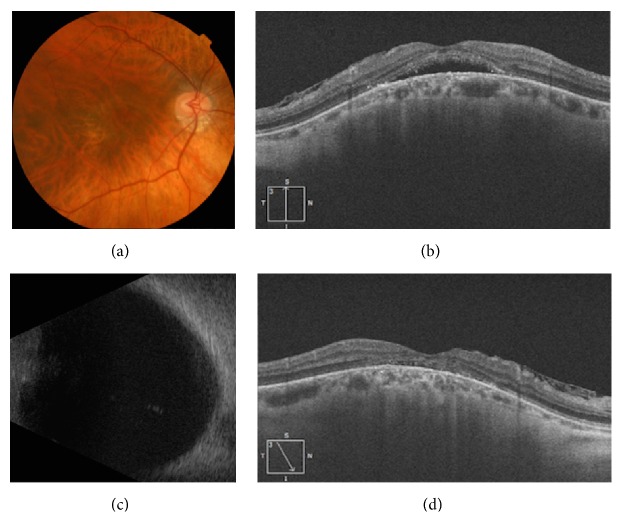
Staphyloma edge with serous retinal detachment before and after treatment with spironolactone. (a) Color photo showing a tilted-disc with a type 5 staphyloma described by Curtin [2], inferior peripapillary atrophy, marked retinal thinning with visible choroidal vessel, but no hemorrhage in the foveal area. (b) Pretreatment OCT image showing serous retinal detachment in the foveal area with associated epiretinal membrane. (c) B-scan ultrasound showing an abnormal ocular wall in the posterior fundus. (d) Posttreatment OCT image showing resolution of the serous retinal detachment in the foveal area with an abnormal ellipsoid line and associated epiretinal membrane.

The study population (Table 1) included nine women and three men, all with bilateral involvement of either a staphyloma edge or a dome-shaped macula. Three patients had dome-shaped maculas (Figure 1), and one eye in each of the three had SRD (Table 1). Nine of the patients had staphyloma margins (Figure 2), and among them there were 11 eyes with SRD (Table 1). The mean age was 48.4 years (range 32–64 years).

Twenty-three eyes had decreased visual acuity at the initial visit and one eye presented with metamorphopsia. The mean spherical equivalent was −4.9 diopters (D) (range: −21.0–+0.50). The mean axial length was 26.93 mm (range: 25.43 mm–31.65 mm). The BCVA before treatment was 0.406 ± 0.324 LogMAR. After treatment it was 0.421 ± 0.354 LogMAR (*P* = 0.489). Therefore spironolactone had no significant effect on BCVA during the period of this study. The mean CRT before treatment was 323.9 ± 78.6 *μ*m. After treatment it had decreased to 291.2 ± 74.5 *μ*m (*P* = 0.010, Figures 1 and 2).

## 4. Discussion

Several hypotheses have been proposed to explain the development of the dome-shaped macula that occurs with myopic staphyloma. Curtin did not specifically describe the dome-shaped macula itself, yet he provided a possible scenario for the development of such structures [2]. Curtin described the septa or steps that are within the staphyloma as ectatics that are smaller than the rest of the staphyloma. Byeon and Chu [11] supported Curtin's theory, describing macular OCT images that had elevations in the combined staphylomas as cited by Curtin. In the first description by Gaucher et al., they proposed that choroid thickening over the macular area and resistance of the sclera to deformation were the probable etiologic factors of this condition [1]. Subsequently, Imamura et al. proposed that a localized scleral thickening in the macular area was the causative factor [12]. This was similar to the recent proposal by Ellabban et al. that the macular bulge resulted from scleral thinning in the parafoveal area near the thicker foveal sclera [13]. Another proposal was that the dome-shaped macula is a protective mechanism that reduces the effects of myopic anisometropia [14], and Mehdizadeh and Nowroozzadeh suggested that it could be secondary to ocular hypotonia, tangential vitreoretinal traction, or scleral invagination due to collapse of the posterior portion of the eyeball [15].

Tilt disc syndrome is characterized by the presence of an ovalized optic disc, situs inversus of the retinal vessels, myopic astigmatism, and visual field defects [16], and it is frequently accompanied by an inferior staphyloma (type V as ranked by Curtin [2]). When the upper margin of this staphyloma crosses the macular area, it may cause a visual deficit because the elevation and hemodynamic alterations cause mechanical changes that lead to the development of macular complications [3, 4].

Coco et al. [3], Nakanishi et al. [4], and Ohsugi et al. [17] have described the complications that can occur with protrusion of the eye wall in patients with a dome-shaped macula or a staphyloma margin. These include SRD, CNV, polypoidal choroidal vasculopathy, RPE atrophy, full-thickness and lamellar macular holes, extrafoveal schisis, and foveoschisis. Among these, SRD is the most frequent complication. While the cause of SRD is not known, several hypotheses have been postulated. These include a complication secondary to abnormal curvature of the macula [1]; mechanical forces and hemodynamic changes [4]; RPE dysfunction [9]; choroidal blood flow obstruction due to a thickened sclera [12]; thickened subfoveal sclera associated with subfoveal choroidal thinning that leads to an abnormal choroidal flow in the fovea with secondary RPE atrophy and damage of the blood-retinal barrier [18]; marked choroidal thinning over the margin of a staphyloma preventing the choroid from removing subretinal fluid [19]; compressive changes of the choroid and choriocapillaris, also called scleral compression maculopathy, that could lead to secondary changes of the RPE and subsequent subretinal fluid accumulation [11]; and RPE detachment that can lead to slow leakage of fluids into the subretinal space [17]. Caillaux et al. reported that SRD is more common when the macular bulge is very high [20]; however, they did not offer any ideas about the pathogenesis of SRD. Recently, Viola et al. proposed that SRD is caused by choroidal vascular changes secondary to excessive scleral thickening, located only within macular protuberance [5]. They also postulated that the appearance of the SRD can change in time in association with the presence or absence of leaking points in the fluorescein angiography, which can lead to spontaneous disappearance of the SRD. In addition to anatomical changes as possible theories for the development of SRD, Dirani et al. recently proposed that a functional alteration in mineralocorticoid pathway could lead to the appearance of SRD [10].

Adequate management of SRD associated with dome-shaped macula or staphyloma edge has never been described. Several therapeutic attempts to resolve SRD conditions have yielded inconsistent or poor results [1, 6–9]. Dirani et al. reported the clinical and anatomical improvement with use of the oral mineralocorticoid antagonist spironolactone in two patients with SRD conditions associated with dome-shaped maculas [10]. Our study demonstrates that spironolactone treatment of SRD conditions associated with dome-shaped maculas or staphyloma edges is a good therapeutic alternative, producing a thickness reduction in the CRT of more than 10%. However, there was no evidence of BCVA improvement.

In our study, there were no reported adverse events related to the use of spironolactone during the monitoring of the patients. It remains an effective and safe drug, although we recommend regular monitoring of potassium serum levels.

This study evaluated prospectively a greater number of patients for a longer period than the original studies of Dirani et al. [10]. However, there were also several limitations. First, we did not perform fluorescein angiography or indocyanine green angiography to confirm or exclude the presence of CNVs. Second, the sample size was small; therefore, the findings should be interpreted with caution. Prospective randomized clinical trials with a control group and targeted biomarkers that are capable of analyzing the therapeutic response to spironolactone are needed to confirm the efficacy of this therapeutic approach.

## 5. Conclusions

There are several anatomical complications that occur in patients with dome-shaped maculas or staphyloma margins. Among these, SRD is the most common. To date it has not been possible to establish the etiology or management of SRD conditions. Oral spironolactone significantly reduced the presence of subretinal fluid; however, functional results are not satisfactory.

## Figures and Tables

**Table 1:** Patient demographics.

Patient	Age	Sex	Group	Staphyloma type	SRD	Systemic disease	Spherical equivalent	AL (mm)
1	43	M	SE	Inferior	RE	None	RE: −1.25	RE: 25.66
							LE: −1.25	LE: 26

2	51	M	SE	Inferior	RE, LE	Porphyria	RE: 0.50	RE: 28.7
							LE: −7.75	LE: 27.3

3	49	F	SE	Inferior	None	Arthrosis	RE: −1.25	RE: 25.72
							LE: −1.00	LE: 25.67

4	32	F	SE	Inferior	RE, LE	Hypothyroidism	RE: −21.00	RE: 31.65
							LE: −17.00	LE: 31.17

5	43	F	SE	Inferior	LE	None	RE: 0.00	RE: 30.10
							LE: 0.00	LE: 30.55

6	58	F	SE	Inferior	RE, LE	SLE	RE: −0.50	RE: 25.95
							LE: −0.25	LE: 26.42

7	36	F	SE	Inferior	RE	Hypothyroidism	RE: −5.50	RE: 27.26
							LE: −17.00	LE: 28.02

8	44	F	SE	Inferior	LE	None	RE: −3.75	RE: 29.06
							LE: −4.75	LE: 29.52

9	64	F	SE	Inferonasal	LE	None	RE: −3.75	RE: 30.77
							LE: −5.50	LE: 26.07

10	56	M	DSM	None	RE	None	RE: −4.00	RE: 25.43
							LE: −5.25	LE: 25.7

11	54	F	DSM	Inferonasal	RE	HBP	RE: −1.00	RE: 25.52
							LE: −0.75	LE: 24.93

12	51	F	DSM	Inferonasal	LE	None	RE: −4.00	RE: 29.16
							LE: −5.50	LE: 30.11

M, male; F, female; SE, staphyloma edge; DSM, dome-shaped macula; SRD, serous retinal detachment; HBP, high blood pressure; SLE, systemic lupus erythematosus; RE, right eye; LE, left eye; AL: axial length.

## References

[1] Gaucher D., Erginay A., Lecleire-Collet A. (2008). Dome-shaped macula in eyes with myopic posterior staphyloma. *American Journal of Ophthalmology*.

[2] Curtin B. J. (1977). The posterior staphyloma of pathologic myopia. *Transactions of the American Ophthalmological Society*.

[3] Coco R. M., Sanabria M. R., Alegría J. (2012). Pathology associated with optical coherence tomography macular bending due to either dome-shaped macula or inferior staphyloma in myopic patients. *Ophthalmologica*.

[4] Nakanishi H., Tsujikawa A., Gotoh N. (2008). Macular complications on the border of an inferior staphyloma associated with tilted disc syndrome. *Retina*.

[5] Viola F., Dell'Arti L., Benatti E. (2014). Choroidal findings in dome-shaped macula in highly myopic eyes: a longitudinal study. *American Journal of Ophthalmology*.

[6] Pardo-López D., Gallego-Pinazo R., Mateo C. (2011). Serous macular detachment associated with dome-shaped macula and tilted disc. *Case Reports in Ophthalmology*.

[7] Tamura N., Sakai T., Tsuneoka H. (2014). Spontaneous resolution of foveal detachment in dome-shaped macula observed by spectral domain optical coherence tomography. *Clinical Ophthalmology*.

[8] Chinskey N. D., Johnson M. W. (2013). Treatment of subretinal fluid associated with dome-shaped macula. *Ophthalmic Surgery Lasers and Imaging Retina*.

[9] Milani P., Pece A., Pierro L., Seidenari P., Radice P., Scialdone A. (2010). Bevacizumab for macular serous neuroretinal detachment in tilted disk syndrome. *Journal of Ophthalmology*.

[10] Dirani A., Matet A., Beydoun T., Mantel I., Behar-Cohen F. (2014). Resolution of foveal detachment in dome-shaped macula after treatment by spironolactone: report of two cases and mini-review of the literature. *Clinical Ophthalmology*.

[11] Byeon S. H., Chu Y. K. (2011). Dome-shaped macula. *American Journal of Ophthalmology*.

[12] Imamura Y., Iida T., Maruko I., Zweifel S. A., Spaide R. F. (2011). Enhanced depth imaging optical coherence tomography of the sclera in dome-shaped macula. *American Journal of Ophthalmology*.

[13] Ellabban A. A., Tsujikawa A., Muraoka Y. (2014). Dome-shaped macular configuration: longitudinal changes in the sclera and choroid by swept-source optical coherence tomography over two years. *American Journal of Ophthalmology*.

[14] Keane P. A., Mitra A., Khan I. J., Quhill F., Elsherbiny S. M. (2012). Dome-shaped macula: a compensatory mechanism in myopic anisometropia?. *Ophthalmic Surgery, Lasers & Imaging Retina*.

[15] Mehdizadeh M., Nowroozzadeh M. H. (2008). Dome-shaped macula in eyes with myopic posterior staphyloma. *American Journal of Ophthalmology*.

[16] Young S. E., Walsh F. B., Knox D. L. (1976). The tilted disk syndrome. *American Journal of Ophthalmology*.

[17] Ohsugi H., Ikuno Y., Oshima K., Yamauchi T., Tabuchi H. (2014). Morphologic characteristics of macular complications of a dome-shaped macula determined by swept-source optical coherence tomography. *American Journal of Ophthalmology*.

[18] Maruko I., Iida T., Sugano Y., Oyamada H., Sekiryu T. (2011). Morphologic choroidal and scleral changes at the macula in tilted disc syndrome with staphyloma using optical coherence tomography. *Investigative Ophthalmology & Visual Science*.

[19] Yamagishi T., Koizumi H., Yamazaki T., Kinoshita S. (2012). Choroidal thickness in inferior staphyloma associated with posterior serous retinal detachment. *Retina*.

[20] Caillaux V., Gaucher D., Gualino V., Massin P., Tadayoni R., Gaudric A. (2013). Morphologic characterization of dome-shaped macula in myopic eyes with serous macular detachment. *American Journal of Ophthalmology*.

